# N^6^-Isopentenyladenosine Enhances the Radiosensitivity of Glioblastoma Cells by Inhibiting the Homologous Recombination Repair Protein RAD51 Expression

**DOI:** 10.3389/fonc.2019.01498

**Published:** 2020-01-14

**Authors:** Giovanna Navarra, Cristina Pagano, Roberto Pacelli, Elvira Crescenzi, Elena Longobardi, Patrizia Gazzerro, Donatella Fiore, Olga Pastorino, Francesca Pentimalli, Chiara Laezza, Maurizio Bifulco

**Affiliations:** ^1^Department of Molecular Medicine and Medical Biotechnology, University of Naples “Federico II,” Naples, Italy; ^2^Department of Advanced Biomedical Sciences, Federico II University School of Medicine, Naples, Italy; ^3^Institute of Endocrinology and Experimental Oncology, Institute of Endocrinology and Experimental Oncology (IEOS), National Research Council (CNR), Naples, Italy; ^4^Section of Pharmacology, Department of Neuroscience University of Naples Federico II, Naples, Italy; ^5^Department of Pharmacy, University of Salerno, Naples, Italy; ^6^Cell Biology and Biotherapy Unit, Istituto Nazionale Tumori, IRCCS, Fondazione G. Pascale, Naples, Italy

**Keywords:** glioblastoma cells, iPA, apoptosis, STAT5a/b, RAD51

## Abstract

Glioblastoma is among the most common malignant brain tumors and has a dismal prognosis due to the poor response to therapeutic regimens such as ionizing radiation and DNA-alkylating agents. In our study, we investigated the radiosensitizing activity of the N^6^-isopentenyladenosine (iPA), an naturally modified adenosine harboring an isopenenyl moiety, which shows antiproliferative effects on glioblastoma cell lines. We observed that co-treatment with ionizing radiation and iPA at micromolar concentration inhibited colony formation and viability of glioblastoma cell lines but not of non-malignant human cells. The combined treatment significantly attenuated the repair of radiation-induced DNA damage by inhibiting both the expression and irradiation-induced foci formation of RAD51, a key player in the homologous recombination repair process, leading to persistent DNA damage, as reflected by an increase of γ-H2AX foci. The radiosensitizing effect relied also on the inhibition of STAT5a/b activation, which is crucial for RAD51 expression, suggesting that iPA modulates the STAT5a/b-RAD51 axis following exposure to ionizing radiation. Overall, these data suggest that iPA, by acting through RAD51 inhibition at the mechanistic level, could function as a promising radiosensitizing agent and warrants further evaluation in prospective clinical trials.

## Introduction

Glioblastoma multiforme (GBM) constitutes the most frequent and aggressive primary brain tumor in adults, characterized by high proliferation rate, invasion into surrounding normal brain tissues and resistance to radio-chemotherapy (1). Current standard therapy consists in surgery followed by radiotherapy in combination with temozolomide (TMZ), a well-tolerated oral chemotherapic agent, which functions as an alkylating agent, by adding methyl groups to purine bases of DNA (2). This therapeutic approach (known as the Stupp protocol) was a breakthrough in GBM treatment improving the 2-years survival to 27%. Interestingly the methylation of the promoter region of the O6-methylguanine-DNA methyltransferase (MGMT) emerged as an important biomarker of increased sensitivity to TMZ, further improving 2-years survival to 47%, in the patients bearing this alteration, leading to a 5-fold increase compared with radiotherapy alone. Indeed, MGMT repairs chemotherapy-induced DNA lesions by removing the alkyl groups added by TMZ, therefore its silencing confers increased sensitivity to TMZ (3). GBM radiotherapy treatment consists of five fractions of 2 Gy at a 6 Gy/min dose rate per week for 6 weeks, the total treatment being ~60 Gy (3). However, most tumors recur at the primary site or in its proximity following radiotherapy, which prompted the search for new therapeutic strategies aimed at increasing tumor radiosensitivity enhancing the efficacy of RT. Most of these approaches focused on drugs able to inhibit the DNA repair process, such as TMZ itself, which acts as a radiosensitizer, inducing DNA damage that includes also highly toxic DNA double-strand breaks (DSBs), and delaying the resolution of phosphorylated histone H2AX (γH2AX) foci, through inhibition of DNA repair (4, 5). N^6^isopentenyladenosine (iPA), a member of the cytokinin family, is a naturally modified nucleoside formed by an adenosine harboring an isopentenyl group at N-6 position. iPA inhibits the growth of human tumor cell lines *in vitro*, inducing the apoptosis of leukemia, sarcoma, myeloma and colon cancer cells (6). It directly interferes with the angiogenic process, which is crucial for tumoral progression (7). Moreover, iPA inhibits the activity of the farnesyl diphosphate synthase (FDPS) enzyme, which catalyzes the synthesis of geranyl pyrophosphate and farnesyl pyrophosphate substrates required for the post-translational modification of proteins involved in signal transduction and cellular proliferation (8–10). We previously described that iPA arrests the proliferation of primary glioblastoma cell lines *in vitro* and *in vivo* via downregulation of epidermal growth factor receptor (EGFR) oncogene-driven pathways (11). A recent study has showed that various enzymes involved in cholesterol biosynthesis, including FDPS, were associated with radioresistance in pancreatic cancer cells. In particular, the knockdown of FDPS, which was overexpressed in human pancreatic cancer tissue, or its pharmacological inhibition through zoledronic acid, radiosensitized pancreatic cancer cells, suggesting that cholesterol synthesis is crucial for radioresistance (12, 13). Consistently, zoledronic acid significantly radiosensitized osteosarcoma cancer cells (13). Lately, we found that GBM express altered levels of the FDPS protein, which abnormally accumulated in all glioma cell lines and in the tumor infiltrated brain of 34 patients (14). So, considering the antitumoral functions of iPA and its ability to inhibit FDPS, we set out to assess whether iPA could act as a radiosensitizer of glioblastoma cancer cells and investigated its biological mechanism in a panel of glioblastoma cancer cells, including U343MG and U87MG (which carry wtp53) and U251 (which carry mutated p53).

## Materials and Methods

### Cells and Culture

Normal Human Astrocytes (NHA) are normal human cells derived from healthy brain tissue, which were grown in astrocyte basal medium (ABM^TM^) supplemented with astrocyte growth medium AGM^TM^ SingleQuots KIT (Lonza). U87MG, U251MG, and U343MG, glioblastoma cancer cell lines, were obtained from CLS Cell Lines Service GmbH (Eppelheim, Germany) cultured in DMEM (Dulbecco's Modified Eagle's Medium) supplemented with 10% heat inactivated fetal bovine serum, 1% L-Glutamine, 1% Sodium Pyruvate, 1% non-essential amino acid (Lonza), and 0.1% plasmocin TM prophylactic (InvivoGen). GBM 18 and GBM 63, primary cell lines of glioblastoma, were cultured in recommended medium DMEM/F-12 Ham (Sigma) supplemented with 15% heat inactivated fetal bovine serum, 2% L-Glutamine, 1% Sodium Pyruvate 1% non-essential (Lonza), 30% D-Glucose, and 1% antibiotic mixture, at 37°C in a humidified atmosphere with 5% carbon dioxide. The adherent primary cultures of brain tumor cells (designated as GBMn) were isolated accordingly to the procedure previously described by our group (13).

### STAT5 Depletion by RNA Interference

STAT5siRNAs (sc-29495) and control-siRNA (sc-37007) were used for transfection U251MG and U343MG cells were seeded in plates at a density of 5 × 10^5^ cells. Both STAT5 and scramble siRNA were delivered into the cell cultures via Lipofectamine RNAi MAX reagent (Invitrogen, CA, USA), according to the manufacturers' instructions. The final concentration of STAT5 and control-siRNA in culture was 1μg. The cells were incubated with the transfection reagents for 48 h, and treated with iPA 1 μM after irradiated at 4 Gy. The cells were then harvested for analysis of protein knockdown via Western Blot analysis.

### Reagents and Abs

N6-isopentenyladenosine (iPA) (Sigma-Aldrich, St. Louis, MO) was dissolved in DMSO and added to cell cultures at the indicated concentration. For Western blot analysis the following antibodies were used: anti-RAD51, anti-pCHK1 (S345), rabbit anti-pCHK2 (T68), anti-p-ATM (S1981), anti-p-BRCA1 (S1524), anti-p-ATR (S428), anti-p-AKT (S473), anti-PARP, anti- p-JAK2 (Tyr 1007/1008), anti-JAK2, anti-NF-κB p65 (D14E12), and anti-Caspase-3 were purchased from Cell Signaling Technology (Danvers, MA), anti-CHK2, anti-STAT5 a/b, anti-H2AX, anti-γ-H2AX (Ser139), anti-β-actin, anti-BRCA1, anti-p-STAT5a/b (Tyr 694/699), anti-p-p38 (Tyr182) were purchased from Santa Cruz Biotechnology (Dallas, TX), anti-CHK1 from Abcam (Cambridge, UK), anti-BCL-2 and anti-p38 from Sigma-Aldrich Inc. (St Luis, MO). For fluorescence microscopy anti-RAD51 (Cell Signaling Technology, Danvers, MA), anti-γ-H2AX (Santa Cruz Biotechnology Dallas, TX) and Alexa Fluor 488 donkey anti-rabbit IgG (Jackson ImmunoResearch, Cambridge, UK) and DyLight 594 goat anti-mouse IgG (Abcam, Cambridge, UK) were used. STAT5a/b-siRNA and scramble-siRNA were purchased from Santa Cruz Biotechnology (Dallas, TX).

### Clonogenic Survival Assay

U343, U251, U87 cells were treated with or without iPA 1 μM for 48 h before irradiation. Irradiation was delivered by 6 MV X ray of a linear accelerator with a dose rate of 200 monitor units for minute and doses of 2 Gy, 4 Gy, and 6 Gy. Post 24 h to irradiation treatment, the cells were then trypsinized, counted, seeded in 6-well plates (1 × 10^3^ cells/plate) and were grown for to 14 days, allowing the surviving cells to produce macroscopic colonies each consisting of 50 or more cells. The colonies were washed with phosphate-buffered solution (PBS), fixed with a fixing solution (Acetic acid/methanol 1:10) for 15 minutes at room temperature (RT), and stained with 0.5% crystal violet solution in 20% MeOH and incubate at RT for 15 min to 2 h. Colonies containing ≥50 cells were counted for each well and the number of colonies corresponding to each radiation dose averaged. The plating efficiency (PE) was calculated as the number of colonies observed/the number of cells plated; the surviving fraction (SF) was calculated as follows: colonies counted/cells seeded X (PE/100). The sensitizer enhancement ratio (SER) was calculated as “dose with radiation alone/dose with radiation+drug for the same biological effect.” If the SER is greater than one, then the addition of the drug is functioning as a radiosensitiser. If the SER is less than one, then the drug is a radioprotector. The experiment was performed in duplicate and repeated 3 times.

### Western Blot Analysis

For analysis of protein levels, cells were grown in p60 tissue culture plates at a density of 5 × 10^5^ cell/cm^2^, treated with iPA for 48 h and subsequently irradiate at 4 Gy. At 24 h after irradiation, cells were washed with PBS, harvested and lysed 30 min with ice-cold RIPA lysis buffer (50 mM Tris-HCl, 150 mM NaCl, 0.5% Triton X-100, 0.5% deoxycholic acid, 10 mg/mL leupeptin, 2 mM phenylmethylsulfonyl fluoride, and 10 mg/ml aprotinin containing protease and phosphatase inhibitors (purchased from Sigma). After being quantified using the Protein Assay Dye Reagent Concentrate (Biorad) and boiled for 5 min in Laemmli buffer 4× (Bio-Rad), equal amounts of protein samples were separated by SDS-PAGE and transferred onto a nitrocellulose membrane (AmershamTM ProtranTM, GE Healthcare Life science, Germany). Membranes were blocked with 5% skim milk powder or 5% BSA in Tris-buffered saline containing 0,1% Tween-20 (TBST) for 1 h at RT and incubated overnight at 4°C with the specific antibodies. Next day, the membrane was incubated with appropiate secondary antibodies (Biorad) for 1 h at RT and subsequently blots were revealed by chemiluminescence (ECLTM Prime Western Blotting Detection Reagents, Amersham, GE Healthcare, Buckinghamshire, UK). Total extracts were normalized by using an anti- β-actin antibody.

### Immunofluorescence Staining of γ-H2AX and Rad51

Cells cultured in 24 well plates with coated glass coverslips were pretreated with 1 μM iPA for 48 h or not, and then irradiated to 4-Gy or not. After treatment, cells were fixed with 3,7% paraformaldehyde for 15 min, permeabilized with 0.2% Triton X-100 for 5 min and blocked with 0,4% bovine serum albumin (BSA, Sigma) in PBS for 15 min at RT. For Rad51 and γ-H2AX foci visualization, samples were stained 1 h with primary antibody against either Rad51 and γ-H2AX followed by incubation with Alexa Fluor 488 (Jackson Immuno Research) and Dylight 594 (Abcam), respectively, for 45 min, in the dark at RT. Subsequently, cell nuclei were counterstained using Hoechst-33258 and coverslips were mounted on glass slides. Cells were visualized and images were captured using confocal fluorescence microscope (Leica).

### Flow Cytometry Analysis

The evaluation of the apoptosis of U343 and U251 glioma cell lines was conducted by anti-human Annexin V and PI staining (Dojindo Molecular Technologies). Briefly, cells grown in p60 tissue culture plates at a density of 5 × 10^5^ cell/cm^2^ in EMEM or DMEM supplemented, treated with 1 μM iPA for 48 h and subsequently irradiate at 4 Gy. At 72 h after irradiation cells were harvested with trypsin and washed in PBS, resuspended in Annexin V binding buffer (10 mMHepes/NaOH, Ph 7; 140 mM NaCl; 2.5 mM CaCl_2_), stained with Annexin V-FITC for 20 min at RT and then with PI at RT for additional 15 min in the dark. The cells were acquired by flow cytometer within 1 h after staining. At least 10,000 events were collected and the data were analyzed by BD Accuri C6 Software. The cells showing positive surface annexin V staining without cell-permeable PI were identified as apoptotic cells. The apoptotic rate is an indicator of apoptosis that calculates the proportion of cells that have undergone apoptosis with respect to the total number of seeded cells. For cell cycle analysis, 24, 48, and 72 h after treatment with iPA 1 μM and radiation 4 Gy, the cells were harvested, washed twice with ice-cold PBS, fixed in 70% cold ethanol and kept at −20°C overnight. The cells were stained with PI (50 μg/ml) in the presence of RNase at 37°C for 30 min at RT in the dark. The stained cells were the subjected to analysis with a flow cytometer and cell cycle distribution were analyzed by BD Accuri C6 Software.

### Quantitative Real-Time PCR

Total RNA was isolated using the NucleoSpin RNA II kit (Macherey-Nagel, Mountain View, CA, USA). Complementary DNA (cDNA) was transcribed using SuperScript II Reverse Transcriptase (Invitrogen), starting from 1 μg of high pure RNA. RAD51 gene expression profile was evaluated with specific primer sets and using SsoFast EvaGreen reagents(Bio-Rad). RAD51 primers (PrimePCR™ PreAmp for SYBR® Green Assay: RAD51, Human Bio-Rad, qHsaCED00421333); β2-microglobulin (primers: Fw, CCTGAATTGCTATGTGTCTGGG, Rev, AATGCGGCATCTTCAAACCTC) was used as housekeeping gene. qRT-PCR protocol was: a pre-heating step for 3 min at 95°C, 40 cycles at 95°C for 10 s and 60° for 30 s and last end-step at 65°C for 10 s. Results were analyzed with 2^−ddCt^ method (15).

### Statistical Analysis

It was performed by using the GraphPad prism 6.0 software for Windows (GraphPad software). For each type of assay or analysis, data obtained from multiple experiments are calculated as mean ± SD and analyzed for statistically significant using the 2-tailed Student *t-test* or ANOVA following by Bonferroni correction for multiple comparisons. *P* > 0.05 were considered significant. ^*^P < 0.05, ^**^P < 0.01, and ^***^P < 0.001.

## Results

### iPA Enhanced the Radiotherapy Effects in Glioblastoma Cancer Cells

**Figure 1:**
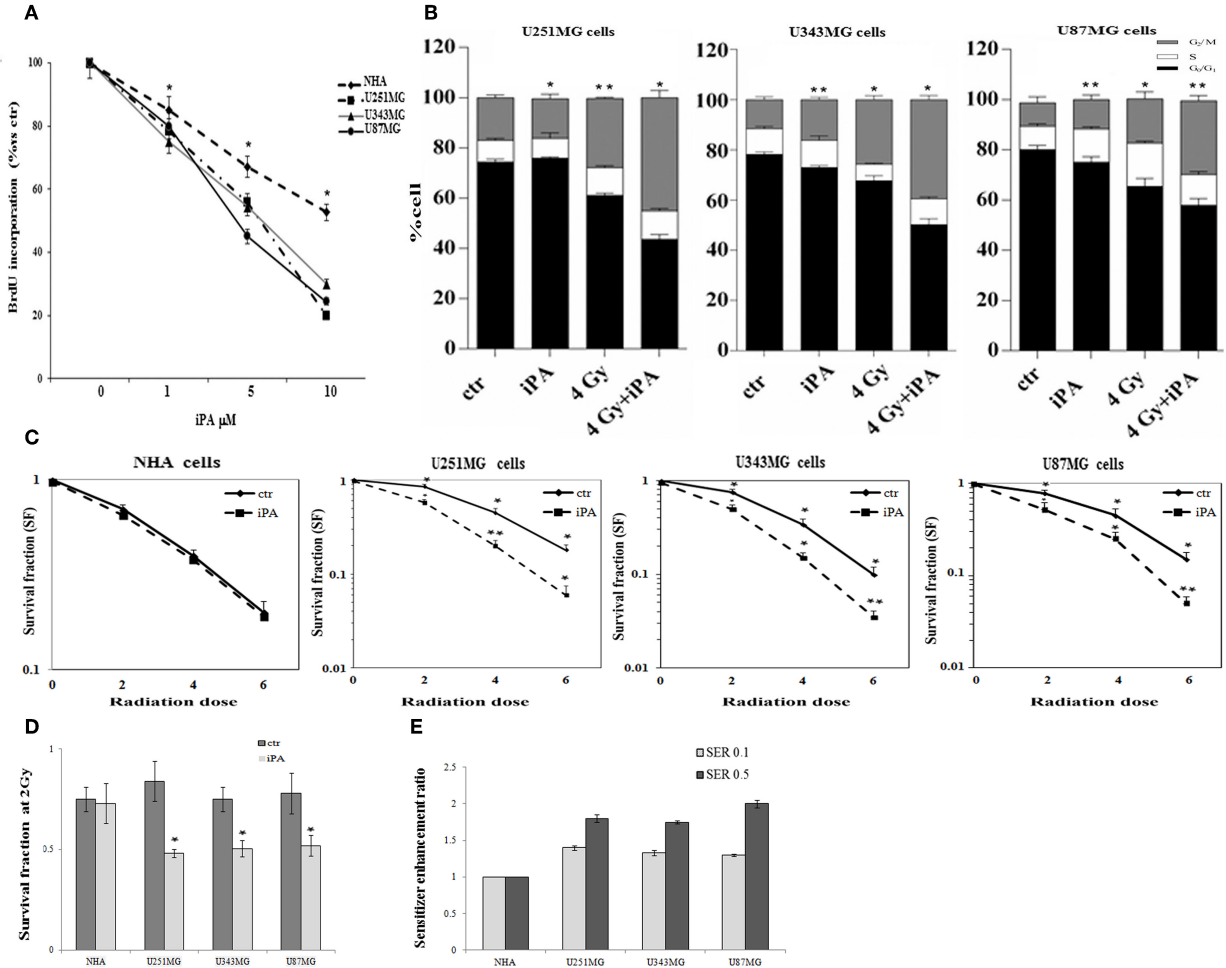
Effect of iPA on glioblastoma cell proliferation. **(A)** Glioblastoma cells were cultured for 48 h in the presence of the indicated concentrations (0–10 μM) of iPA, before the assessment of BrdU incorporation. Results are expressed as means ± SD of five independent experiments performed in triplicate and reported as percentage vs. the untreated control (ANOVA, *p < 0.05 vs. control). **(B)** Distribution of U251MG, U343MG, and U87MG cells in the different phases of the cell cycle in cells pretreated with iPA for 48 h followed from irradiation at 4-Gy as compared to IR alone. Histograms show the percentage of cells in each phase of the cell cycle. Data analyzed for statistical significant using the 2-tailed Student t-test or ANOVA following by Bonferroni correction for multiple comparisons. Results are representative of three experiments performed in duplicate, and expressed as mean ± SD (ANOVA *p < 0.05). Effect of iPA on glioblastoma cells radiosensitivity: **(C)** Clonogenic survival curves for NHA, U251MG, U343MG, and U87MG cells were exposed to 1 μM of iPA or DMSO for 48 h and irradiated with increasing doses of X-rays, followed by an additional 24 h post-irradiation incubation in iPA containing medium. The results shown represent the average of 3 independent experiments. **(D)** The survival fraction at 2-Gy (SF2) of human glioblastoma cells lines treated with iPA or control (DMSO) for 48 h pre-irradiation. Values are the mean survival fraction ± SD of at least 3 independent experiments. Error bars represent the standard error. *indicates *p* < 0.05. **(E)** Sensitizer enhancement ratios (SER) was calculated at 10 or 50% cell survival (0.1 or 0.5). Distribution of U251MG, U343MG, and U87MG cells in the different phases of the cell cycle in cells pretreated with iPA for 48 h followed from irradiation at 4-Gy as compared to IR alone. Histograms show the percentage of cells in each phase of the cell cycle. Data analyzed for statistical significant using the 2-tailed Student t-test or ANOVA following by Bonferroni correction for multiple comparisons. Results are representative of 3 experiments performed in duplicate, and expressed as mean ± SD (ANOVA *p < 0.05; **p < 0.01).

**Figure 2:**
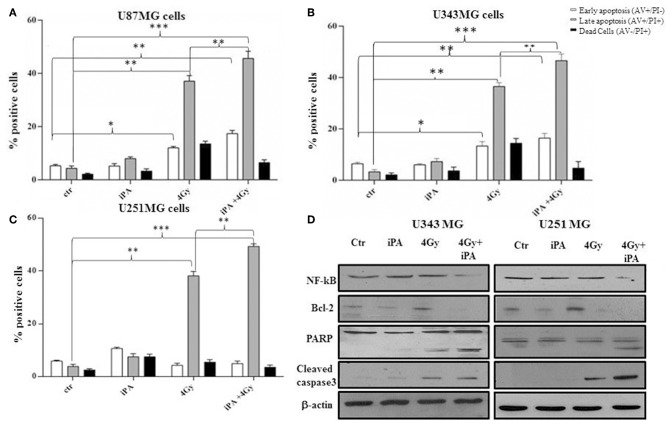
Effect of iPA in combination with IR on integrity of human glioblastoma cells. **(A–C)** Flow cytometric analysis of Annexin V and propidium iodide (PI) double staining in glioblastoma cells. Histograms show the total percentage of early (Annexin V-positive cells/PI-negative cells) and late apoptotic events (Annexin V/PI-double positive cells) as well as necrotic cells (Annexin V-negative cells/PI-positive cells). Results are representative of five experiments performed in duplicate and expressed as mean ± SD (ANOVA *p < 0.05, **p < 0.01, ***p < 0.001). **(D)** Expression of typical apoptosis markers, cleaved caspase-3, cleaved PARP, Bcl2, and NF-kB based on western blotting. β-actin was used as loading control.

We, previously, described that iPA treatment inhibited the proliferation of glioblastoma cancer cells in a dose- and time-dependent manner, with an IC50, as measured by inhibition of BrdU incorporation, was in the range of ~7.5 μM at 48 h (11, 15). Here, we examined the effect of iPA on the radiosensitivity of U251MG cells (p53mut), U343MG (p53wt), and U87MG (p53wt) glioblastoma cells in comparison to a normal counterpart, the Normal Human Astrocytes (NHA) cell line, through a colony formation assay. All cells were pretreated with 1 μM of iPA for 48 h and exposed to ionizing radiation (IR) at 2, 4, and 6-Gy. We used iPA at the dose of 1 μM for 48 h because this concentration had a low effect on cell proliferation, as assessed by BrdU incorporation assay and cell cycle analysis (Figure 1A). In all three tested glioblastoma cells lines, pretreatment with iPA significantly increased sensitivity to IR, as reflected by the cell decreased ability to form colonies, independently of their p53 status in comparison to NHA cell line. We observed that the clonogenicity of the glioblastoma cells treated with iPA+IR was reduced in an IR-dose dependent manner (Figure 1C). The degree of radiosensitization was measured by confronting the surviving fractions at the radiation dose of 2-Gy (SF2) and by calculating the sensitizer enhancement ratios (SER). As a result, iPA pretreatment reduced the SF2 values of glioblastoma cells compared with the cells treated with IR-alone, whereas, iPA had not significant effect on NHA cells (Figure 1D). In particular, the combined treatment reduced the SF2 values of U251MG cells from 84.5% ± 2.2 to 61.2% ± 0.5, of U343MG from 75.1% ± 1.5 cells to 50.5% ± 1.5 and of U87MG cells from 78.2% ± 2.1 to 52.5% ± 1.2. The SER values (Figure 1E) indicated that iPA pretreatment significantly increased radiation sensitivity in glioblastoma cell lines. To investigate the molecular mechanism of radiosensitization induced by iPA, we analyzed, through flow cytometer, the cell cycle profiles of the glioblastoma cells following pretreatment with iPA 1 μM for 48 h before irradiation at the single dose of 4-Gy. As shown in Figure 1B, we observed that iPA alone had no effect on the cell cycle distribution compared with untreated cells after 48 h, while IR alone induced G2/M phase arrest in glioblastoma cells as expected. When IR were combined with 1 μM iPA, cell accumulation in the G2/M phase increased suggesting that iPA pretreatment could enhance cell cycle arrest. In order to ascertain whether the iPA+IR combination was able to trigger apoptosis, we analyzed cell death by Annexin V and propidium iodide double staining (Figures 2A–C). Notably, 72 h after irradiation, the apoptotic rate of the combined treatment was 49.3% ± 1.8 for U251 cells, of 46.6% ± 2.5 for U343MG cells and of 45.6 ± 2.2 for U87MG cells remarkably higher than that of glioblastoma cells treated with radiation alone, which was 38.1 ± 2.2 for U251MG cells, 36.5 ± 1.8 for U343MG cells and 37.5 ± 1.2 for U87MG cells, respectively. At the molecular level, we found in U251MG and U343MG cell lines, 24 h after the combined treatment, increased caspase-3 and PARP cleavage with respect to cells treated with IR alone. Moreover, the anti-apoptotic BCL2 protein, which was upregulated by IR treatment, was remarkably downregulated by combined treatment with iPA in both U251MG and U343MG cells. Interestingly, iPA+IR combined treatment reduced also the protein levels of NFkB (Figure 2, Supplementary Information), the activation of which, following DNA damage, emerged as a key mediator of cancer resistance to genotoxic agents (16). Overall, iPA likely lowered the apoptotic threshold of glioblastoma cells acting on different antiapoptotic players.

### iPA Pretreatment Before Irradiation Increased DNA Damage

**Figure 3:**
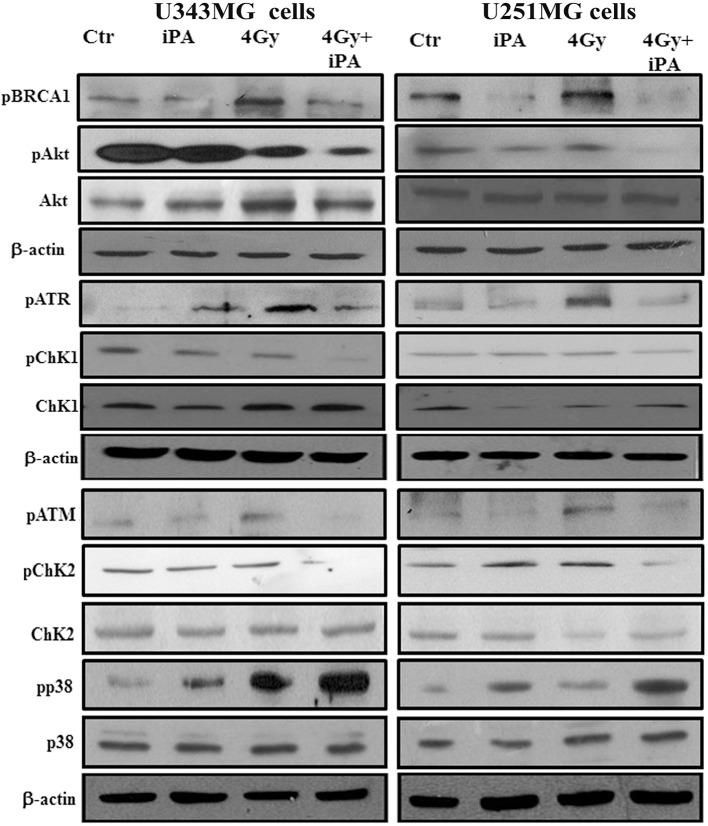
iPA inhibited DNA repair pathway. U343MG cells and U251MG cells were treated with iPA alone for 48 h, or combined with 4 Gy IR. Cell lysates were analyzed by Western blot analysis for DNA repair pathway proteins including pAkt (S473), pCHk1 (S345), pCHK2 (Thr-68), pBRCA1 (Ser-1524), pATM (Ser-1981), and pATR (Ser-428).

**Figure 4:**
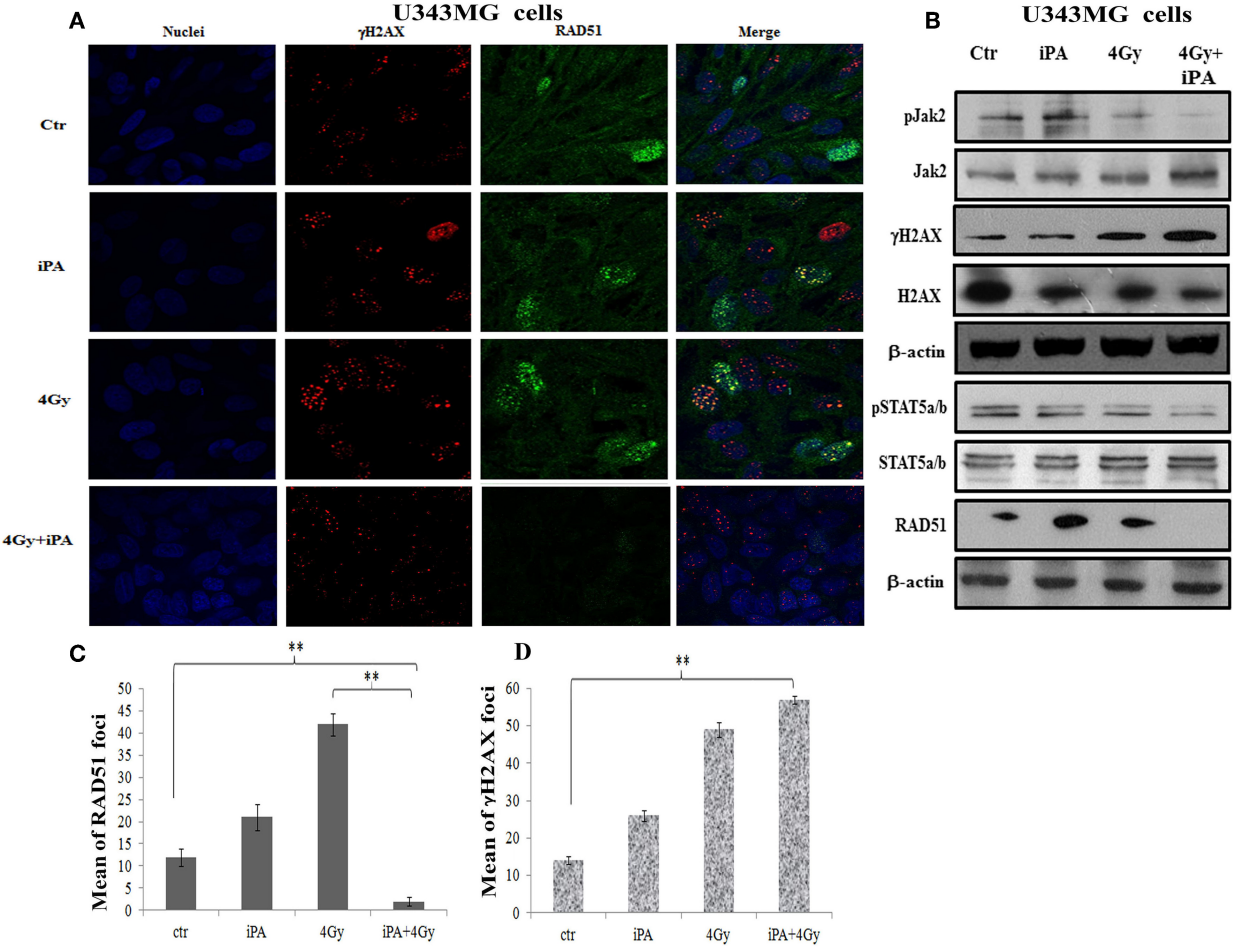
iPA inhibited the RAD51 foci formation of U343MG cells. **(A)** The cells were plated on glass cover slips and pre-treated with iPA for 48 h and then irradiated with 4Gy IR. After 24 h wells were washed and analyzed by confocal microscopy for γH2AX foci (red) and Rad51 foci (green) and Hoechst 33258 for nuclear staining (blue). One representative experiment of a total of three is shown. **(B)** iPA inhibited the protein levels RAD51 via inactivation of Jak2/Stat5a/b signaling pathway. Whole cell extracts treated with or without 4 Gy of IR were collected for Western blot analysis using Jak2, Stat5a/b, RAD51, andγ-H2AX. β-actin was used as an internal loading control. **(C,D)** DNA damage foci and RAD51 foci were counted and plotted as bar graphs. Approximately 200 nuclei for each treatment group were scored in each experiment, and a threshold of 5 foci per cell was considered positive. Values represent the means of 3 experiments ± SD (ANOVA, *p < 0.05, **p < 0.01).

**Figure 5:**
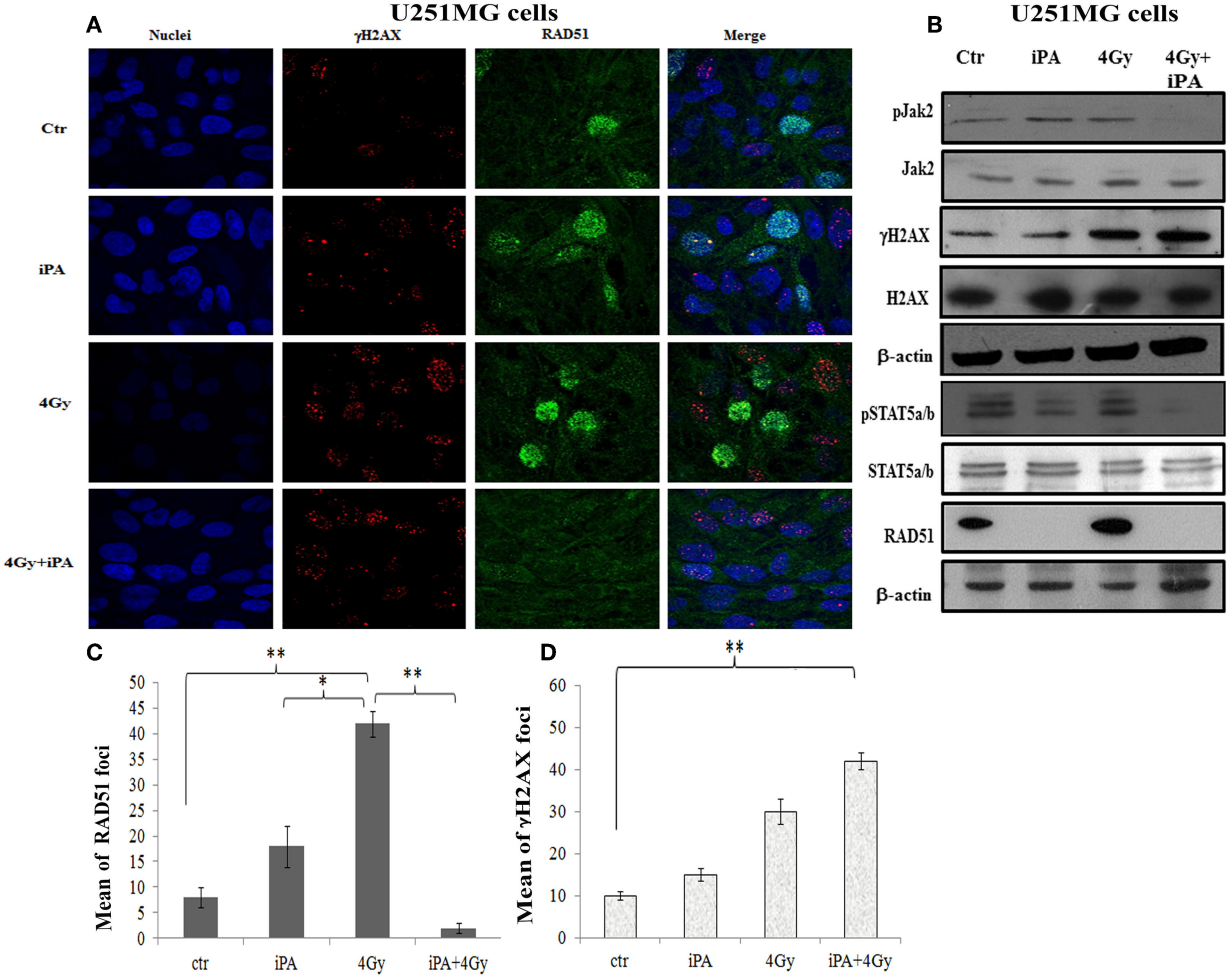
iPA inhibited the RAD51 foci formation of U251MG cells. **(A)** Immunocytochemistry for phosphorylated γH2AX (red), for RAD51 (green) in U251MG cells exposed to radiation in the presence of iPA and analyzed at 24 h after irradiation. Hoechst 33258 used for nuclear staining (blue). **(B)** Whole cell extracts were collected for Western blot analysis using Jak2, Stat5a/b, RAD51, andγ-H2AX. β-actin was used as loading control. **(C,D)** Histograms represent quantification of RAD51 foci and γH2AX foci in U251MG cells exposed to 4-Gy or the iPA/radiation combination. Values represent the means of 3 experiments ± SD; (ANOVA, *p < 0.05, **p < 0.01).

Because glioma radioresistance depends on the preferential activation of the DNA damage checkpoint response and subsequent increase in DNA repair capacity (5), we examined a panel of key DNA damage response (DDR) players by western blotting analysis. Including the effector kinases ATM and ATR; the checkpoint kinases CHK2 and CHK1; BRCA1, which links DNA damage sensing to DDR effectors; the DSB marker γH2AX. We measured phosphorylation of ATM at serine 1981 and CHK2 at threonine 68 in U251MG and U343MG cells after 24 h exposure to radiation alone and to iPA+ 4-Gy IR in comparison to untreated or cells treated with iPA or IR alone (Figure 3, Supplementary Information). We noted that the 4-Gy IR induced a robust phosphorylation of ATM S1981 and CHK2 Thr68 whereas the iPA+IR combined treatment strongly reduced the phosphorylation of both. The combined treatment reduced also the phosphorylation of ATR, CHK1, Akt kinase, and the phosphorylation of BRCA1 compared with cells treated with IR. Conversely, we found an hyperactivation of MAPK p38 in glioblastoma cells treated with iPA+IR compared to cells treated with IR or iPA alone or untreated cells, suggesting the involvement of p38 MAPK in the induction of a G2/M cell cycle checkpoint in response to DNA damage stimuli that induce DSBs (17). Because ATM and ATR phosphorylation are crucial for DSB-induced checkpoint responses and DSB repair (18), these results suggest that iPA+IR treatment could inhibit the repair of IR-induced DSB. For this purpose, we examined the expression levels of the DSB marker γH2AX (19) by western blotting analysis. We found that the iPA+IR combination therapy remarkably increased the protein levels of γH2AX, compared with cells treated with single agents (Figures 4B, 5B, Supplementary Information). Consistently, immunofluorescence analysis of γ-H2AX foci in U343MG and U251MG cells 24 h after exposure to iPA+IR, IR, or iPA alone showed that the combined treatment increased the number of the frequency of γ-H2AX foci positive cells (Figures 4A, 5A). In contrast, iPA alone resulted in the appearance of weak foci 24 h after the treatment. Interestingly, the increase of γH2AX expression levels persisted up to 72 h after irradiation (data not shown), suggesting that the combined treatment delayed DNA damage repair improving the radiotherapy efficacy.

### iPA Downregulated RAD51 Expression in Glioblastoma Cancer Cells by Inhibition Jak2-STAT5a/b Pathway

**Figure 6:**
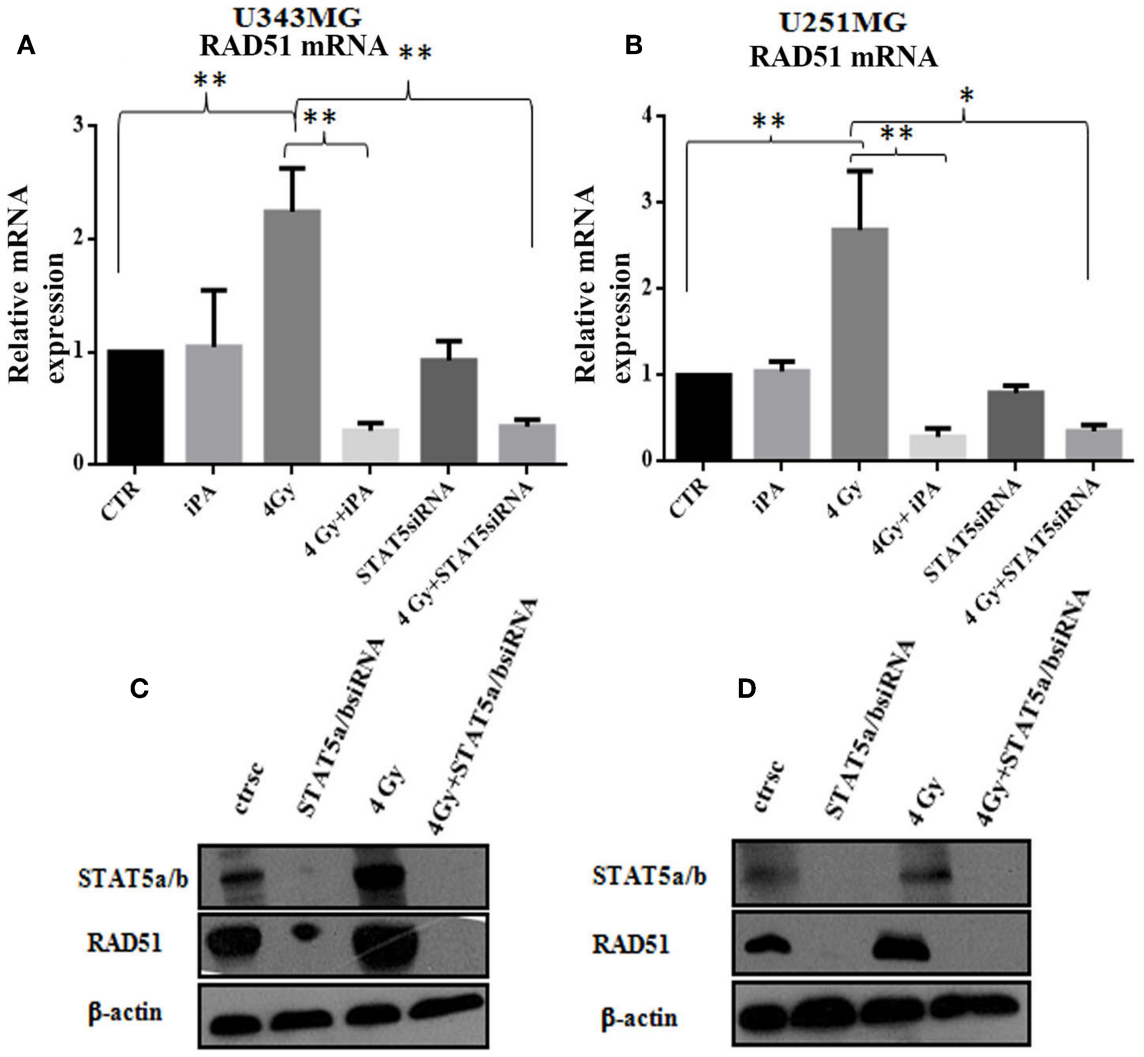
Stat5a/b was inhibited by transfection with of STAT5a/b siRNA (siSTAT5a/b) in U343MG and U251MG. **(A,B)** Rad51 mRNA levels. Cells were exposed to iPA or transfected with STAT5a/b-siRNA for 48 h followed by irradiation at 4Gy and collected for RT-PCR analysis of the Rad51/β2microglobulin mRNA ratios. Each value represents the mean ± SE for five independent experiments. Results are expressed as mean ± SD (ANOVA *p < 0.05, **p < 0.01) compared with control values. **(D,C)** western blot analysis of Rad51 expression in U343MG and U251MG cells transfected with STAT5a/b-siRNA vs. control (scramble-siRNA, ctrsc) for 48 h followed by irradiation at 4 Gy. Effective genetic depletion of Stat5a/b are demonstrated by immunoblotting with anti-STAT5a/b **(C,D)**. β-actin was used as loading control.

DSB repair is achieved through two main pathways, the most faithful of which is the homologous recombination (HR) pathway. HR occurs through a tightly regulated series of events, in which RAD51 recombinase plays a key role (20). Targeting RAD51-dependent repair is known to enhance tumor cell radiosensitivity (21). So, to assess whether RAD51 was involved in the radiosensitizing effect induced by iPA, we evaluated RAD51 levels by western blot and its ability to form foci by immunofluorescence in glioblastoma cells pretreated with iPA 1 μM plus irradiation at 4-Gy in comparison to untreated cells or cells treated with iPA or IR alone. We observed that the IR induced an upregulation of RAD51 protein levels. To confirm that RAD51 is functional in these cells, we, next, examined foci formation and we founded a robust increase in RAD51 foci numbers in response to IR which were inhibited by iPA pretreatment (Figures 4, 5). Then, because STAT5a/b has been connected to DNA repair in chronic myeloid leukemia (CML) and it is a transcriptional regulator of RAD51 (22, 23), we have explored the possibility that STAT5a/b could be a potential target of iPA in glioblastoma cells. Indeed, iPA+IR combined treatment reduced the phosphorylation of STAT5a/b in comparison to untreated cells or cells treated with iPA or IR alone. To dissect further the STAT5 signaling cascade, we evaluated he phosphorylation state of the Janus Kinase (JAK2), the prototypical upstream kinase responsible for STAT5 phosphorylation in glioblastoma cells treated with iPA+IR. Activated p-JAK2 was significantly increased in irradiated glioblastoma cells whereas the iPA+IR combined treatment strongly reduced the JAK2 phosphorylation. Moreover, we investigated the level of RAD51 mRNA in both cancer cell lines after treatment with IPA and in combination with IR. We observed that ionizing radiation induced an up-regulation of RAD51 mRNA levels in U251MG and U343MG cells (Figures 6A,B), consistent with the increase of RAD51 at protein level. Because IR can upregulate the expression of RAD51, we next tested whether iPA pretreatment could counteract this effect of IR. The cells were first treated with iPA then with IR and the level of RAD51 expression was measured. In contrast to the elevation of RAD51 mRNA in these cells that were treated with IR alone, iPA pretreatment effectively attenuated the up-regulation of RAD51 mRNA induced by IR (Figure 6), indicating that iPA was able to overcome the upregulation of RAD51 induced by IR. Because iPA may affect Jak2/Stat5 pathways involved in the transcription of RAD51 we next depleted STAT5a/b in glioblastoma cells by RNAi and tested the mRNA levels of RAD51. As shown in Figures 6C,D, (Supplementary Information) STAT5 expression could be efficiently silenced by siRNA specifically targeting STAT5 in both cell lines. As expected, the depletion of STAT5 led to a significant reduction of mRNA level of RAD51 in cells treated with IR. These results suggest that the radiosensitizing effect of iPA in glioblastoma cells was primarily mediated by the downregulation of transcription of RAD51mRNA dependent on inhibition of STAT5a/b activation.

### iPA Increased the Radiosensitivity of the Primary Glioblastoma Cells

**Figure 7:**
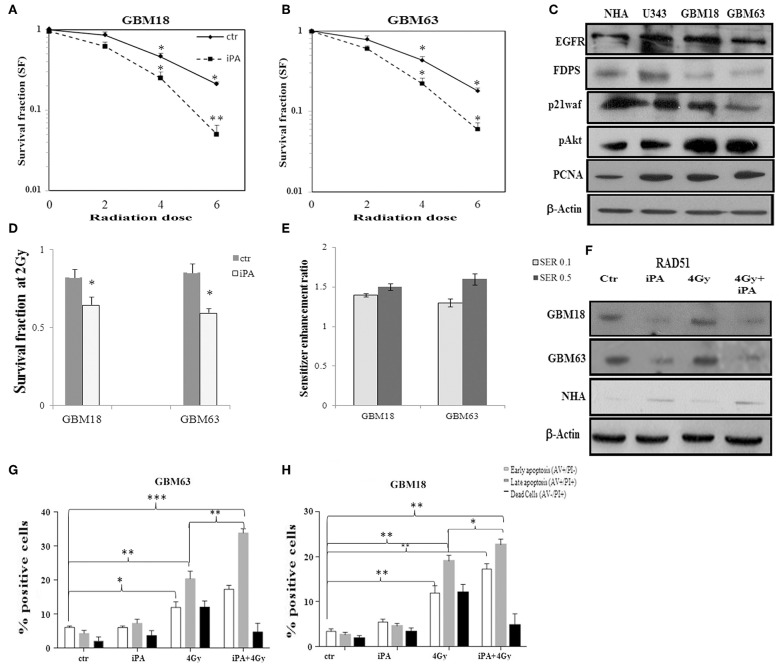
Effect of iPA on tumor cell radiosensitivity on primary glioblastoma cells. **(A,B)** Clonogenic survival curves for GBM18 and GBM63 cells were exposed to 1 μM of iPA or DMSO for 48 h and irradiated with increasing doses of X-rays, followed by an additional 24 h post-irradiation incubation in iPA contaning medium. The results shown represent the average of 3 independent determinations. **(D)** The survival fraction after 2-Gy (SF2) of human primary glioblastoma cells. Values are the mean survival fraction ± SD of at least 3 independent experiments (ANOVA *p < 0.05). **(E)** Sensitizer enhancement ratios (SER) were calculated at 10% or 50% cell survival (0.1 or 0.5) **(C)** Representative Western blot showing the basal protein levels of EGFR, p21waf, p-AKT, AKT, FDPS, and PCNA in the primary human glioblastoma cells. **(F)** Western blot showing the RAD51 protein levels in GBM18, GBM63, and in NHA cells. β-actin was used as loading control. **(G,H)** Flow cytometric analysis of Annexin V and propidium iodide (PI) double staining in GBM18 and GBM63 cells. Histograms show the total percentage of early (Annexin V-positive cells/PI-negative cells) and late apoptotic events (Annexin V/PI-double positive cells) as well as necrotic cells (Annexin V-negative cells/PI-positive cells). Results are representative of 5 experiments performed in duplicate and expressed as mean ± SD (ANOVA *p < 0.05, **p < 0.01, ***p < 0.001).

Based on these results, we tested the antitumor effectiveness of the iPA/radiation combination in two primary cell lines derived from fresh tumoral masses of two selected patients with glioblastoma multiforme, the GBM18 and GBM63 cells. These primary glioblastoma cells were positive for the expression of EGFRwt, p21waf, and FDPS compared to U343 glioma cell line and normal human astrocytes representing their healthy counterpart. These primary cells were, also, characterized by high levels of the associated oncogenic signal transduction proteins as pAKT and PCNA compared to NHA and, as expected, oncogenic potential was conserved in U343 glioblastoma cell line (Figure 7C, Supplementary Information). The primary GBM cells were pretreated for 48 h with iPA 1 μM after exposure to IR at 2, 4, and 6-Gy and then after 24 h subjected to the clonogenic assay. In the primary glioblastoma cells, pretreatment with iPA significantly increased sensitivity to IR, as reflected by the cell decreased ability to form colonies (Figures 7A,B). We observed that the clonogenicity of the glioblastoma cells treated with iPA+IR was reduced in an IR-dose dependent manner (Figures 7A,B). The degree of radiosensitization was measured by confronting the surviving fractions at the radiation dose of 2-Gy (SF2) and by calculating the sensitizer enhancement ratios (SER). As a result, iPA pretreatment reduced the SF2 values of glioblastoma cells compared with the cells treated with IR-alone (Figure 7D) and enhanced the radiosensitivity with a SER at 0.5 of 1.5 for GBM18 cells and 1.6 for GBM63 cells (Figure 7E), in comparison to the cells treated with IR alone. In order to verify whether iPA increased radiotoxicity was associated with cell apoptosis, we analyzed cell death analysis by Annexin V and propidium iodide double staining. We observed that after 72 h from irradiation with 4-Gy dose, the apoptotic rate of iPA+IR was 33.7 ± 2.5% for GBM18 cells, of 22.5 ± 1.2% for GBM63 cells while in cells treated with radiation alone it was 20.5 ± 1.82 for GBM18 cells and 18.2 ± 1.2 for GBM63 as compared to untreated cells (5.8 ± 0.5% for GBM18 cells and 2.1 ± 0.5% GBM63 cells) and treated with iPA alone (6 ± 1.2% for GBM18 cells and 4.2 ± 1.2% for GBM63 cells) (Figures 7G,H). Finally, we analyzed the RAD51 expression levels in primary GBM cells with respect to NHA. GBM18 and GBM63 cell exposure to iPA1 μM (Figure 7F, Supplementary Information) did not increase the RAD51 protein levels as compared with untreated cells which showed high RAD51 protein levels; conversely, radiation increased RAD51 protein levels compared to iPA-treated cells, consistently with the results obtained in the established glioblastoma cell lines. When iPA-pretreated cells were irradiated, RAD51 protein levels were reduced as compared with the radiation alone and untreated cells, suggesting that iPA 1 μM reduced the radiation-stimulated increase in RAD51 protein levels. Unlike the primary GBM cells, iPA+IR induced the RAD51 protein levels in the NHA cells, confirming the lack of radiosensitizing effect of iPA on normal human astrocytes.

## Discussion

### iPA Enhanced the Human Glioma Cancer Cells Radiosensitivity

Radiotherapy in combination with chemotherapy, represent a key component in GBM treatment (24). However, the cytotoxicity to normal tissue and tumor radioresistance are fatal problems in radiotherapy. Sensitizing cancer cells to DNA-damaging agents, such as IR, can be achieved by directly targeting key proteins of the DDR, including the ATM and ATR kinases (25) or RAD51 recombinase (26). The current study is the first demonstration that iPA enhanced at low concentration the radiosensitivity of glioblastoma cells by downregulating RAD51, a key player in HR repair, leading to impairment of a prompt DDR. iPA exhibits promising antiproliferative effects against various cancer cells including glioblastoma cells (6–9, 11) but the radiosensitization efficacy of iPA in glioblastoma cells that exhibit high radioresistance was previously overlooked. In this study, we observed that iPA at the low 1 μM concentration in combination with low dose of ionizing radiation (4-Gy) inhibited the colony forming ability of glioblastoma cells.

For most patients a median dose of 54 (range 40–60) Gy is used but patients with a poor general condition receive a palliative dose of 30-Gy in 10 fractions (3-Gy per fraction) (27). For this reason, we used a single dose of 4-Gy in combination with iPA. Clonogenic assay revealed that NHA were not radiosensitized by iPA in combination with IR and this correlated with the lack of a cytotoxic effect of iPA on these normal cells (11). Moreover, the SF2 and SER _0.1_ and SER_0.5_values suggest that radiosensitizing glioblastoma cells with iPA could have several benefits, such as lowering the doses of both agents to restrict side effects while enhancing the therapeutic window of radiotherapy. iPA complies these criterions by inducing arrest in G2/M phase, the most radiosensitive phase of the cell cycle (28), promoting caspase 3-mediated signaling and programmed cell death, reducing NF-kB levels (29), inhibiting radiation-induced AKT pro-survival signaling and enhancing DNA DSB formation (30). Indeed, we observed that ionizing radiation combined with iPA induced high levels of γ-H2AX protein, a key marker for DSB lesions, besides an increased number of γ-H2AX foci after 24 h. Moreover, the combined treatment delayed the resolution of γ-H2AX proteins, suggesting that iPA maintains DNA damage enhancing the radiosensitivity of glioblastoma cells.

### iPA Inhibited HR DNA Repair Pathway and Downregulated Rad51 Expression in Human Glioma Cancer Cells

The cancer radiosensitivity is primarily determined by the capacity of cancer cells to repair radiation-induced DNA damages (31). In this study we found that iPA inhibited the repair of IR-induced DNA DSBs, and that the iPA effects affected the HR pathway. Our data, indeed, show that iPA+IR combined treatment decreased the phosphorylation of the ATM and CHK2 kinases, crucial players of the HR repair of DNA DSBs, implying the inhibition of this pathway (25). ATM represents a fulcrum of the DDR having roles in both cell cycle control and DNA repair, whereas CHK2 is a main phosphorylation target of ATM and an important intermediary of cell cycle checkpoints following ionizing radiation (32). More, signaling by ATR/CHK1 also enhances repair by inducing DNA repair proteins and recruiting repair factors to the damage site. We also showed that iPA+IR decreased the phosphorylation of ATR, CHK1, BRCA1, and Akt while inducing the phosphorylation of p38 MAPK. Attenuation of ATM-CHK2 and ATR-CHK1 determined the inhibition of BRCA1 phosphorylation, which plays a role in to the DNA repair processes (33). BRCA1 and BRCA2 are positioned with RAD51 to sites of DNA damage and replication forks and a functional BRCA1 is required for RAD51 foci formation (34). Activation of p38 MAPK induced by radiation alone, or in combination with iPA, might trigger the nuclear accumulation of p38 MAPK inducing a G2/M cell cycle checkpoint in response to DNA damage stimuli that induce DSBs (35). Numerous studies have shown that several cancer cells, including GBM cells as compared to normal cells, have higher levels of RAD51, demonstrating its involvement in resistance to IR and that RAD51 downregulation determined the radiosensitivity (36–39). In this study, we ascertained that the exposure of both GBM cell lines to ionizing radiation resulted in an increase in RAD51 protein and mRNA levels. whereas, iPA pretreatment of glioblastoma cells reduced radiation induced RAD51 protein and mRNA levels and nuclear foci formation. Moreover, we observed that iPA alone reduced RAD51 protein levels but not mRNA levels into U251MG cells, suggesting a different modulation of RAD51 protein in these cells. These data indicate that iPA increases tumor cell killing induced by radiation and suggest that the mechanism involves the reduction in RAD51 protein and mRNA levels. Unlike GBM cell lines, we observed that in NHA cells radiation did not induce RAD51 protein levels while iPA either alone or in combination with radiation induced the RAD51 protein levels. These data suggest that RAD51 expression levels could be differently regulated in GBM and NHA and that iPA could have a radioprotective effect on normal tissue, although this stimulates further investigations.

### iPA Suppressed mRNA Rad51 Levels Through Inactivation of JAK2/STAT5a/b Pathway

Maranto et al. (23) have described that JAK2–STAT5a/b signaling promotes DNA repair through HR in prostate cancer by upregulating RAD51. Conversely, inhibition of STAT5a/b depletes RAD51 levels, disrupting HR DNA repair, and sensitizing prostate cancer to radiation. This is consistent with the presence of a STAT5a/b responsive element in the regulatory region of the *RAD51*gene (23). Based on these evidences, we checked iPA effect on JAK2-STAT5a/b phosphorylation state. We observed that iPA+IR inhibited JAK2 and STAT5a/b (Y694/696) phosphorylation, affecting the transcriptional activity of STAT5. We, indeed, observed that genetic knockdown of STAT5 by specific siRNA significantly suppressed Rad51 levels in glioblastoma cancer cells, indicating that STAT5a/b regulates the Rad51 expression levels. Moreover, it worth noting that the tyrosine phosphorylation (Y696) of STAT5a/b via a JAK2-dependent mechanism promoting DNA-binding activity is triggered by RhoA GTP binding protein, the activity of which is dependent on prenylation (40). We have described that iPA inhibits FDPS causing in the cell a reduction of farnesylpyrophosphate (FPP) and of its downstream product geranylgeranylpyrophosphate (GGPP) both essential for the activity of the prenylated proteins, including RhoA (41) involved also in the regulation of DNA repair pathways, particularly in early repair. These evidences suggest a possible role of RhoA in the mechanism of action of iPA, further upstream to the STAT5a/b-RAD51 axis herein identified. Overall, these results represent another evidence that the inhibition of JAK2-STAT5a/b signaling by iPA might provide a possible adjuvant therapy for irradiation of glioblastoma, enhancing the sensitivity to radiation and reducing radiation-induced damage to the neighboring tissues. In conclusion, our results from the primary glioblastoma cells (GBM18 and GBM63 cells) indicate that iPA effect on RAD51 expression levels is not restricted to an immortalized cell line suggesting that at least a subset of primary cells use the signaling pathway found in glioblastoma cancer cell line. The combination therapy based on iPA and subsequent X-irradiation could be considered a promising alternative to the standard GBM treatment since the current radiotherapy of glioblastoma occurs at very high doses (56–60-Gy) (42, 43). Based on these observations, further experimentation with animal models and clinical trials should be explored.

## Data Availability Statement

The datasets generated for this study are available on request to the corresponding author.

## Author Contributions

GN, CP, EC, EL, DF, and OP performed the experiment and helped in experiment designing, data analysis, and statistical analysis. PG, FP, CL, RP, and MB helped with manuscript writing, editing, and proofreading. DF and OP Performed siRNA STAT5a/b, transfection assay ans relatives Western blots.

### Conflict of Interest

The authors declare that the research was conducted in the absence of any commercial or financial relationships that could be construed as a potential conflict of interest.

## References

[1] Hambardzumyan D Bergers G. Glioblastoma: defining tumor niches. Trends Cancer. (2015) 1:252–65. 10.1016/j.trecan.2015.10.009 27088132 PMC4831073

[2] Stupp R Mason WP van den Bent MJ Weller M Fisher B Taphoorn MJB. Radiotherapy plus concomitant and adjuvant temozolomide for glioblastoma. N Engl J Med. (2005) 352:987–96. 10.1056/NEJMoa043330 15758009

[3] Uhm JH1 Porter AB Treatment of glioma in the 21st century: an exciting decade of postsurgical treatment advances in the molecular era. Mayo Clin Proc. (2017) 92:995–1004. 10.1016/j.mayocp.2017.01.010 28578786

[4] Kil WJ Cerna D Burgan WE Beam K Carter D Steeg PS. *In vitro* and *in vivo* radiosensitization induced by the DNA methylating agent temozolomide. Clin Cancer Res. (2008) 14:931–8. 10.1158/1078-0432.CCR-07-1856 18245557

[5] Bindra RS Chalmers AJ Evans S Dewhirst M. GBM radiosensitizers: dead in the water…or just the beginning? J Neurooncol. (2017). 134:513–2. 10.1007/s11060-017-2427-7 28762004

[6] Bifulco M Malfitano AM Proto MC Santoro A Caruso MG Laezza C. Biological and pharmacological roles of N6-isopentenyladenosine: an emerging anticancer drug. Anticancer Agents Med Chem. (2008). 8:200–4. 10.2174/187152008783497028 18288922

[7] Pisanti S Picardi P Ciaglia E Margarucci L Ronca R Giacomini A. Antiangiogenic effects of N6-isopentenyladenosine, an endogenous isoprenoid end product, mediated by AMPK activation. FASEB J. (2014) 28:1132–44. 10.1096/fj.13-238238 24265487

[8] Laezza C Notarnicola M Caruso MG Messa C Macchia M Bertini S. N6-isopentenyladenosine arrests tumor cell proliferation by inhibiting farnesyl diphosphate synthase and protein prenylation. FASEB J. (2006) 20:412–8; 10.1096/fj.05-4044lsf 16507758

[9] Ciaglia E Pisanti S Picardi P Laezza C Malfitano AM D'Alessandro A. N6-isopentenyladenosine, an endogenous isoprenoid end product, directly affects cytotoxic and regulatory functions of human NK cells through FDPS modulation. J Leukoc Biol. (2013) 94:1207–19. 10.1189/jlb.0413190 23847096

[10] Scrima M Lauro G Grimaldi M Di Marino S Tosco A Picardi P. Structural evidence of N6-isopentenyladenosine as a new ligand of farnesyl pyrophosphate synthase. J Med Chem. (2014) 57:7798–803. 10.1021/jm500869x 25184810

[11] Ciaglia E Abate M Laezza C Pisanti S Vitale M Seneca V. Antiglioma effects of N6-isopentenyladenosine, an endogenous isoprenoid end product, through the downregulation of epidermal growth factor receptor. Int J Cancer. (2017) 140:959–72. 10.1002/ijc.30505 27813087

[12] Souchek JJ Baine MJ Lin C Rachagani S Gupta S Kaur S. Unbiased analysis of pancreatic cancer radiation resistance reveals cholesterol biosynthesis as a novel target for radiosensitisation. Br J Cancer. (2014) 111:1139–49. 10.1038/bjc.2014.385 25025965 PMC4453840

[13] Kim EH Kim MS Lee KH Koh JS Jung WG Kong CB. Zoledronic acid is an effective radiosensitizer in the treatment of osteosarcoma. Oncotarget. (2016) 7:70869–880. 10.18632/oncotarget.12281 27765919 PMC5342595

[14] Abate M Laezza C Pisanti S Torelli G Seneca V Catapano G. Deregulated expression and activity of Farnesyl Diphosphate Synthase (FDPS) in glioblastoma. Sci Rep. (2017) 7:14123. 10.1038/s41598-017-14495-6 29075041 PMC5658376

[15] Livak KJ Schmittgen TD Analysis of relative gene expression data using real-time quantitative PCR and the 2(-Delta Delta C(T)) methods. Methods. (2001) 25:402–8. 10.1006/meth.2001.1262 11846609

[16] Ciaglia E Laezza C Abate M Pisanti S Ranieri R D'Alessandro A. Recognition by natural killer cells of N6-isopentenyladenosine-treated human glioma cell lines. Int J Cancer. (2018) 142:176–90. 10.1002/ijc.31036 28884474

[17] Hoesel B Schmid JA. The complexity of NF-κB signaling in inflammation and cancer. Mol Cancer. (2013) 12:86. 10.1186/1476-4598-12-86 23915189 PMC3750319

[18] Bulavin DV Higashimoto Y Popoff IJ Gaarde WA Basrur V Potapova O. Initiation of a G2/M checkpoint after ultraviolet radiation requires p38 kinase. Nature. (2001) 411:102–7. 10.1038/35075107 11333986

[19] Blackford AN Jackson SP. ATM, ATR, and DNA-PK: the trinity at the heart of the DNA damage response. Mol Cell. (2017) 66:801–17. 10.1016/j.molcel.2017.05.015 28622525

[20] Rothkamm K Barnard S Moquet J Ellender M Rana Z Burdak-Rothkamm S. DNA damage foci: meaning and significance. Environ Mol Mutagen. (2015) 56:491–504. 10.1002/em.21944 25773265

[21] Helleday T Petermann E Lundin C Hodgson B Sharma RA Nat Rev Cancer. (2008) 8:193–204. 10.1038/nrc2342 18256616

[22] Ward A Khanna KK Wiegmans AP. Targeting homologous recombination, new pre-clinical and clinical therapeutic combinations inhibiting RAD51. Cancer Treat Rev. (2015) 41:35–45. 10.1016/j.ctrv.2014.10.006 25467108

[23] Maranto C Udhane V Hoang DT Gu L Alexeev V Malas K. STAT5A/B blockade sensitizes prostate cancer to radiation through inhibition of RAD51 and DNA repair. Clin Cancer Res. (2018) 24:1917–31. 10.1158/1078-0432.CCR-17-2768 29483142 PMC6036914

[24] Hasselbach L Haase S Fischer D Kolberg HC Sturzbecher HW. Characterisation of the promoter region of the human DNA-repair gene Rad51. Eur J Gynaecol Oncol. (2005) 26:589–98. 16398215

[25] Khosla D. Concurrent therapy to enhance radiotherapeutic outcomes in glioblastoma. Ann Transl Med. (2016) 4:2–9. 10.3978/j.issn.2305-5839.2016.01.25 26904576 PMC4740000

[26] Maréchal A Zou L. DNA damage sensing by the ATM and ATR kinases Cold Spring HarbPerspect Biol. (2013) 5:012716. 10.1101/cshperspect.a012716 24003211 PMC3753707

[27] Huang F. Inhibition of homologous recombination in human cells by targeting RAD51 recombinase. J. Med Chem. (2012) 55:3011–20. 10.1021/jm201173g 22380680

[28] Hingorani M Colley WP Dixit S Beavis AM Hypofractionated radiotherapy for glioblastoma: strategy for poor-risk patients or hope for the future? Br J Radio. (2012) 1017:e770–81. 10.1259/bjr/83827377 PMC3487099 22919020

[29] Pawlik TM Keyomarsi K. Role of cell cycle in mediating sensitivity to radiotherapy. Int J Radiat Oncol Biol Phys. (2004) 59:928–42. 10.1016/j.ijrobp.2004.03.005 15234026

[30] Kang KH Lee KH Kim MY Choi KH. Caspase-3-mediated cleavage of the NF-kappa B subunit p65 at the NH2 terminus potentiates naphthoquinone analog-induced apoptosis. J Biol Chem. (2001) 276:24638–44. 10.1074/jbc.M101291200 11320092

[31] Liu Q Turner KM Alfred Yung WK Chen K Zhang W. Role of AKT signaling in DNA repair and clinical response to cancer therapy. Neuro Oncol. (2014) 16:1313–23. 10.1093/neuonc/nou058 24811392 PMC4165418

[32] Gerweck LE Vijayappa S Kurimasa A Ogawa K Chen DJ. Tumor cell radiosensitivity is a major determinant of tumor response to radiation. Cancer Res. (2006) 66:8352–55. 10.1158/0008-5472.CAN-06-0533 16951142

[33] Smith J Tho LM Xu N Gillespie DA. The ATM-Chk2 and ATR-Chk1 pathways in DNA damage signaling and cancer. Adv Cancer Res. (2010) 108:73–112. 10.1016/B978-0-12-380888-2.00003-0 21034966

[34] Burdak-Rothkamm S Rothkamm K McClelland K Prise KM. BRCA1, FANCD2 and Chk1 are potential molecular targets for the modulation of a radiation-induced DNA damage response in bystander cells. Cancer Lett. (2015) 356:454–61. 10.1016/j.canlet.2014.09.043 25304378 PMC4259524

[35] Cousineau I Abaji C Belmaaza A. BRCA1 regulates RAD51 function in response to DNA damage and suppresses spontaneous sister chromatid replication slippage: implications for sister chromatid cohesion, genome stability, and carcinogenesis. Cancer Res. (2005) 65:11384–91. 10.1158/0008-5472.CAN-05-2156 16357146

[36] Munshi A Ramesh R. Mitogen-activated protein kinases and their role in radiation response. Genes Cancer. (2013) 4:401–8. 10.1177/1947601913485414 24349638 PMC3863336

[37] Raderschall E Stout K Freier S Suckow V Schweiger S Haaf T. Elevated levels of Rad51 recombination protein in tumor cells. Cancer Res. (2002) 62:219–25. 11782381

[38] Saydam O Saydam N Glauser DL Pruschy M Dinh-Van V Hilbe M. HSV-1 amplicon-mediated post-transcriptional inhibition of Rad51 sensitizes human glioma cells to ionizing radiation. Gene Ther. (2007) 14:1143–51. 10.1038/sj.gt.3302967 17495946

[39] Russell JS Brady K Burgan WE Cerra MA Oswald KA Camphausen K. Gleevec-mediated inhibition of Rad51 expression and enhancement of tumor cell radiosensitivity. Cancer Res. (2003) 63:7377–83. 14612536

[40] Welsh JW Ellsworth RK Kumar R Fjerstad K Martinez J Nagel RB. Rad51 protein expression and survival in patients with glioblastoma multiforme. Int J Radiat Oncol Biol Phys. (2009) 74:1251–55. 10.1016/j.ijrobp.2009.03.018 19545791

[41] Benitah SA Valerón PF Rui H Lacal JC. STAT5a activation mediates the epithelial to mesenchymal transition induced by oncogenic RhoA. Mol Biol Cell. (2003) 14:40–53. 10.1091/mbc.e02-08-0454 12529425 PMC140226

[42] Osaki JH Espinha G Magalhaes YT Forti FL Modulation of RhoA GTPase activity sensitizes human cervix carcinoma cells to γ-radiation by attenuating DNA repair pathways. Oxid Med Cell Longev. (2016) 2016:6012642 10.1155/2016/6012642 PMC4662998 26649141

[43] Cabrera AR Kirkpatrick JP Fiveash JB Shih HA Koay EJ Lutz S. Radiation therapy for glioblastoma: executive summary of an American society for radiation oncology evidence-based clinical practice guideline. Pract Radiat Oncol. (2016) 6:217–25. 10.1016/j.prro.2016.03.007 27211230

