# Supplementary material for: N6-Isopentenyladenosine Enhances the Radiosensitivity of Glioblastoma Cells by Inhibiting the Homologous Recombination Repair Protein RAD51 Expression
Source: Front Oncol. 2020 Jan 14;9:1498. doi: 10.3389/fonc.2019.01498 (PMC6971108; doi:10.3389/fonc.2019.01498)

Supplementary Information

N^6-isopentenyladenosine enhances the radiosensitivity of glioblastoma cells by inhibiting of the homologous recombination repair protein RAD51 expression

Giovanna Navarra[1], Cristina Pagano[1], Roberto Pacelli[2], Elvira Crescenzi[3], Elena Longobardi[4], Patrizia Gazzerro[5], Donatella Fiore[5],Olga Pastorino[1], Francesca Pentimalli[6], Chiara Laezza[3*], Maurizio Bifulco[1*].

[1]Department of Molecular Medicine and Medical Biotechnology, University of Naples "Federico II", [2]Department of Advanced Biomedical Sciences, Federico II University School of Medicine, Naples, Italy, [3]Institute of Endocrinology and Experimental Oncology, IEOS CNR,[4]Section of Pharmacology, Dept. of Neuroscience University of Naples Federico II, [5]Department of Pharmacy, University of Salerno, [6]Cell Biology and Biotherapy Unit, Istituto Nazionale Tumori, IRCCS, Fondazione G. Pascale, Napoli 80131, Italy. *Maurizio Bifulco and Chiara Laezza jointly supervised this work.

Densitometric analysis of blots in Figure 2D

U343MG cells **U251MG cells**

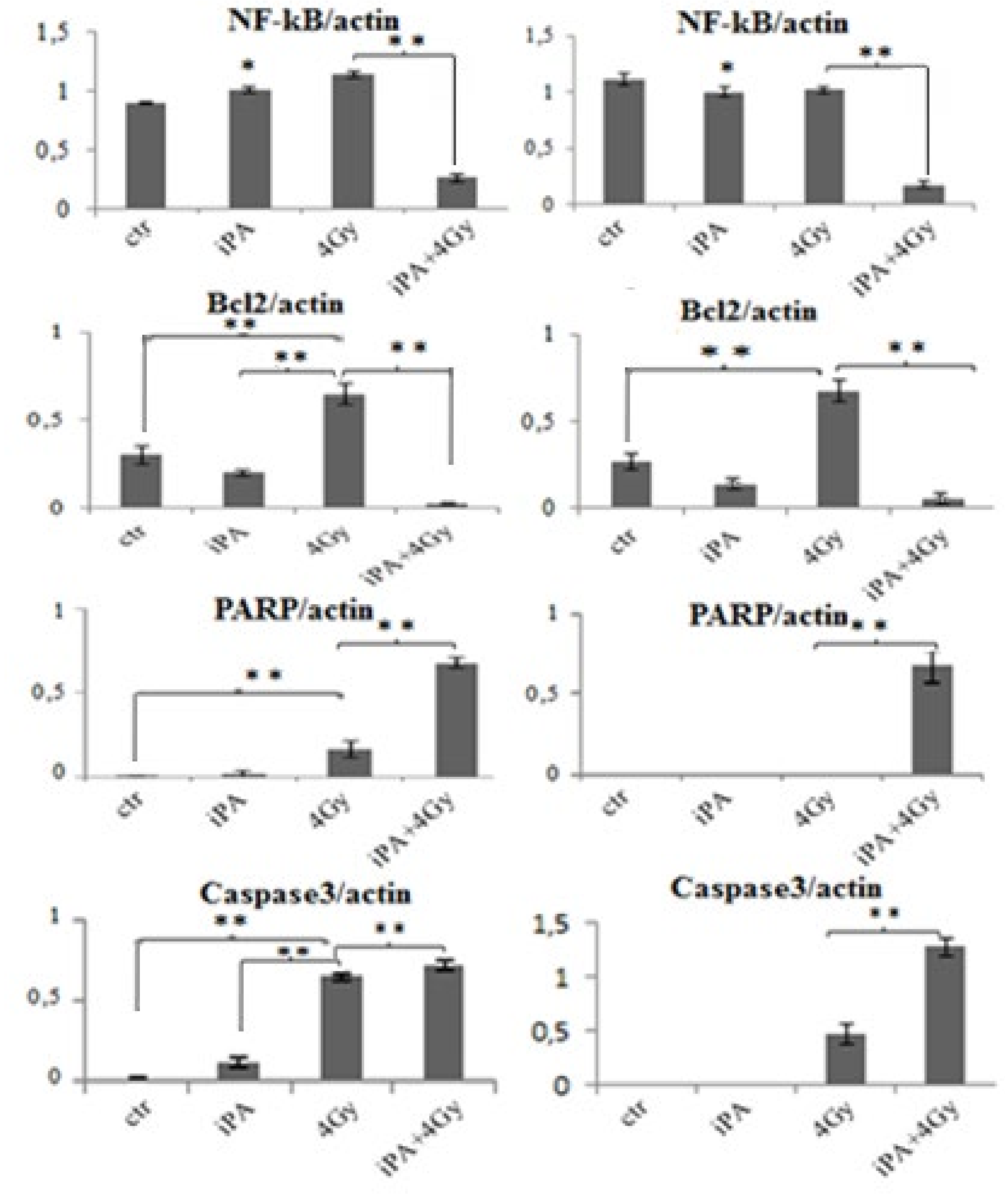

Densitometric analysis of blots in Figure 3

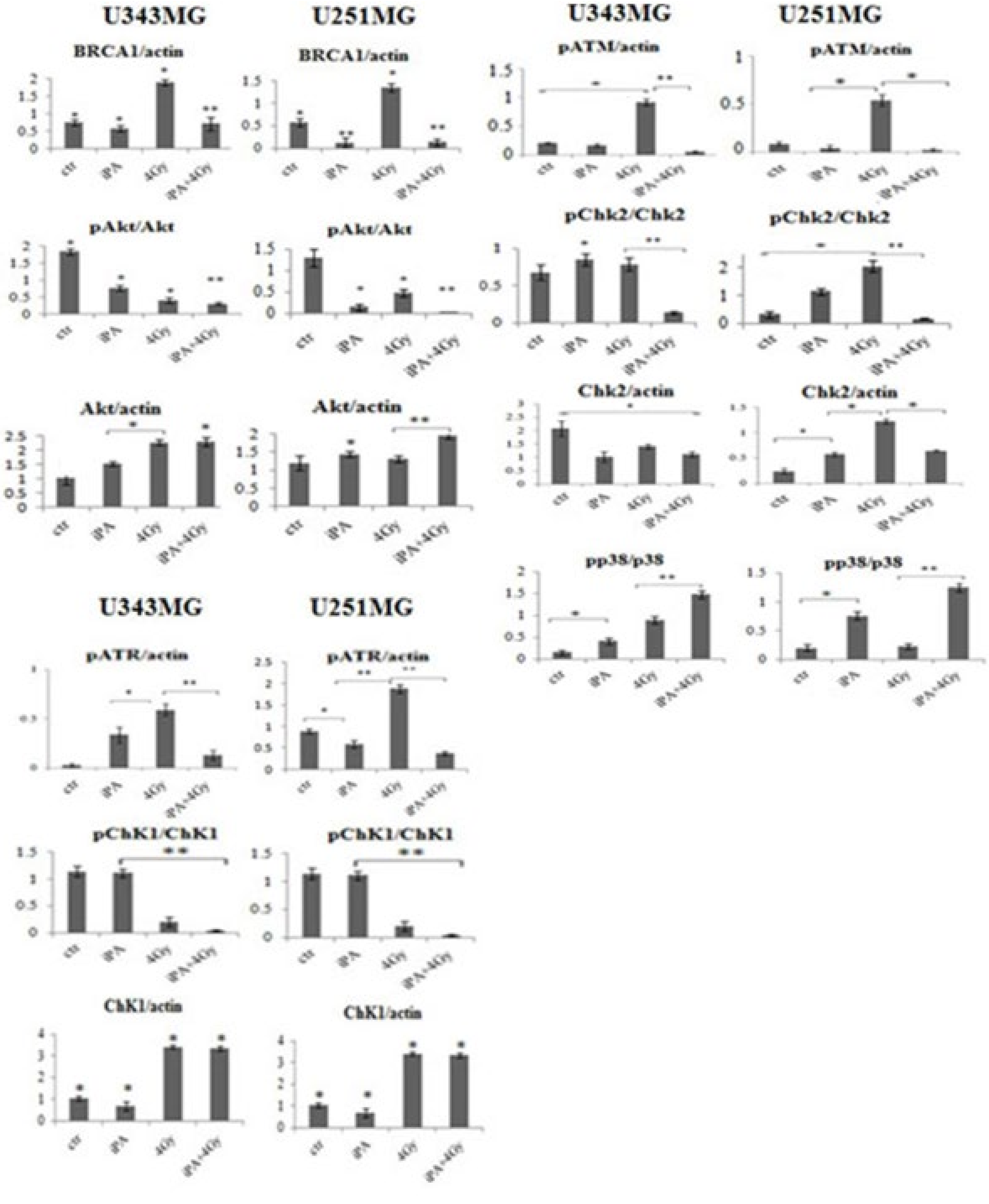

Densitometric analysis of blots in Figure 4B and 5B

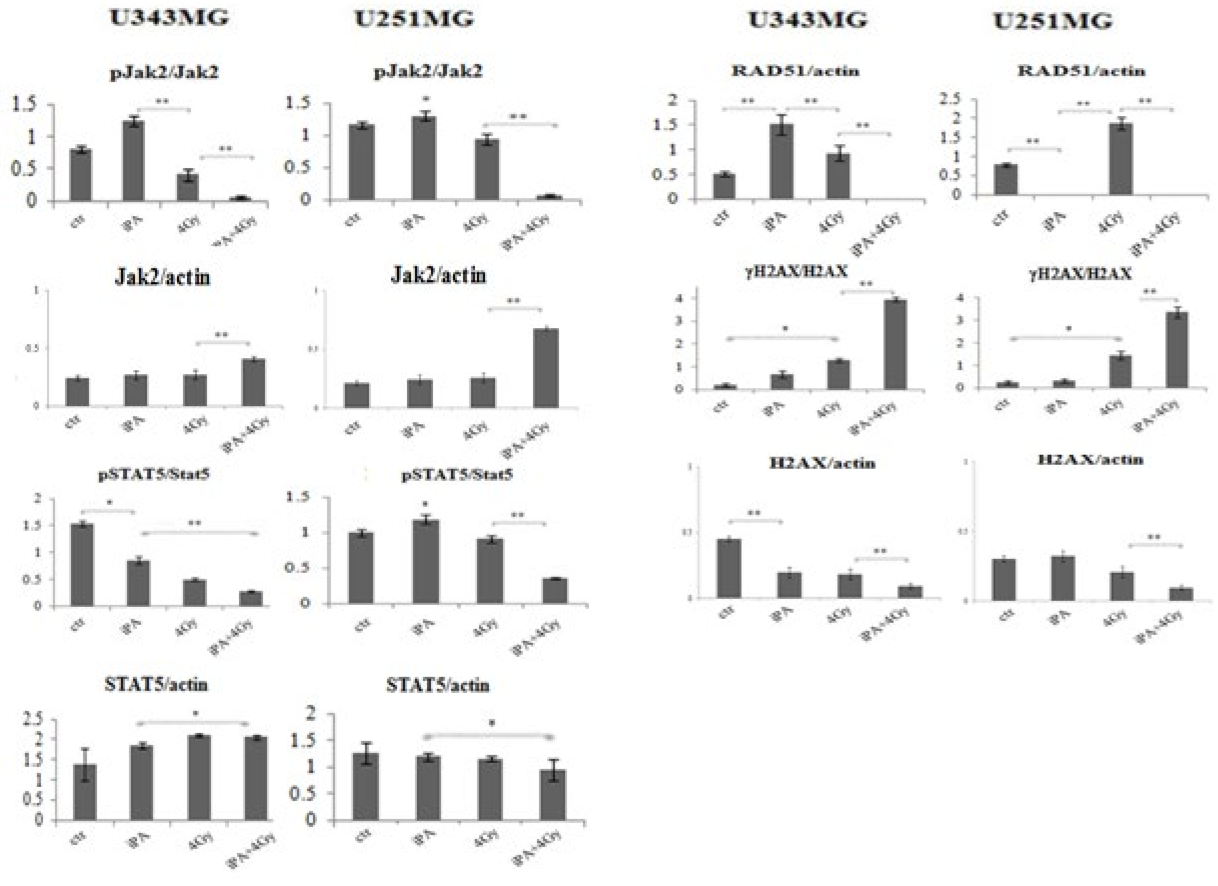

Densitometric analysis of blots in Figure 6C

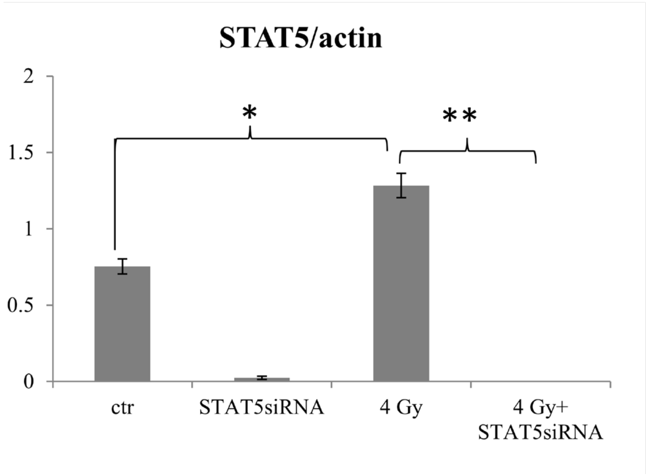

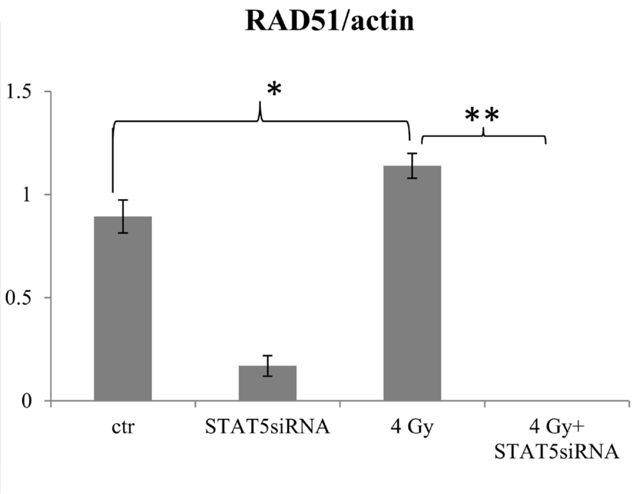

Blots in Figure 6D

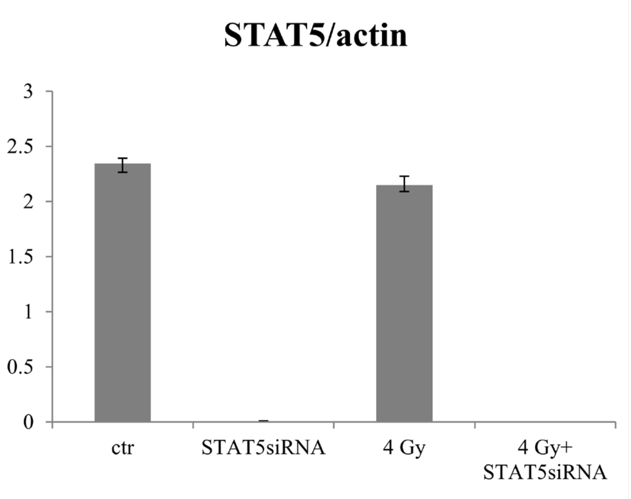

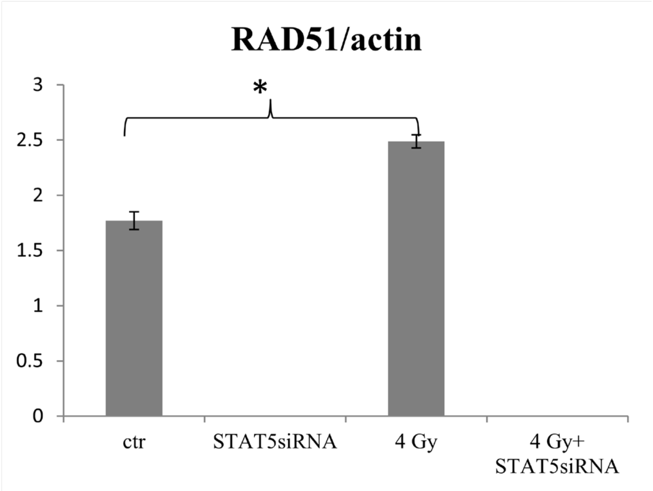

Densitometric analysis of blots in Figure 7C

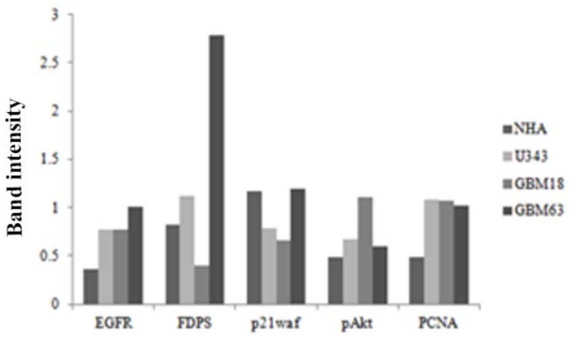

Densitometric analysis of blots of RAD51 in Figure 7F

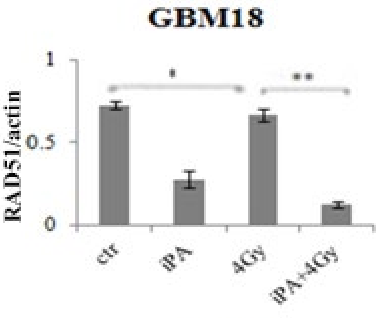

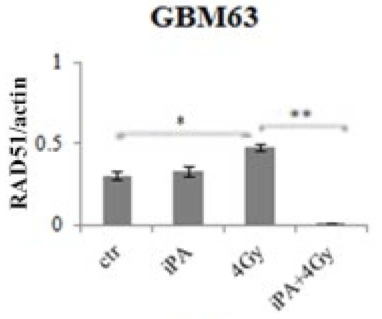

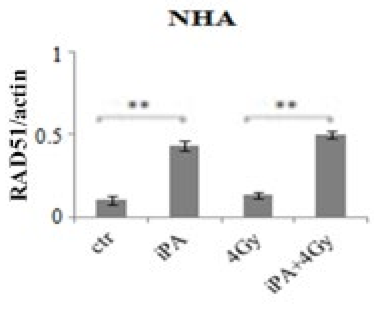

Supplement: Supplementary file 1 [file Data_Sheet_1.pdf]
